# M^3^D: a kernel-based test for spatially correlated changes in methylation profiles

**DOI:** 10.1093/bioinformatics/btu749

**Published:** 2014-11-13

**Authors:** Tom R. Mayo, Gabriele Schweikert, Guido Sanguinetti

**Affiliations:** ^1^IANC, School of Informatics, University of Edinburgh, Edinburgh EH8 9AB and ^2^Wellcome Trust Centre for Cell Biology, University of Edinburgh, Edinburgh EH9 3JR, UK

## Abstract

**Motivation:** DNA methylation is an intensely studied epigenetic mark implicated in many biological processes of direct clinical relevance. Although sequencing-based technologies are increasingly allowing high-resolution measurements of DNA methylation, statistical modelling of such data is still challenging. In particular, statistical identification of differentially methylated regions across different conditions poses unresolved challenges in accounting for spatial correlations within the statistical testing procedure.

**Results:** We propose a non-parametric, kernel-based method, M^3^D, to detect higher order changes in methylation profiles, such as shape, across pre-defined regions. The test statistic explicitly accounts for differences in coverage levels between samples, thus handling in a principled way a major confounder in the analysis of methylation data. Empirical tests on real and simulated datasets show an increased power compared to established methods, as well as considerable robustness with respect to coverage and replication levels.

**Availability and implementation:** R/Bioconductor package M^3^D.

**Contact:** G.Sanguinetti@ed.ac.uk

**Supplementary information:** Supplementary data are available at *Bioinformatics* online.

## 1 Introduction

DNA methylation is an epigenetic mark associated with many fundamental biological processes of direct clinical relevance, such as imprinting, retrotransposon silencing and cell differentiation (Gopalakrishnan *et al*., 2008; Laurent *et al*., 2010). Methylation occurs when a methyl group is attached to a cytosine. In mammals, methylation is observed predominantly in the CpG context, and, consequently, studies tend to focus on these loci. The canonical understanding is that methylation of CpG regions in promoter regions (CGIs) is associated with gene silencing; however, recent studies have shown that CpG methylation correlates with gene expression in a more complex and context-dependent manner (Varley *et al*., 2013). Methylation profiles are altered in many diseases, most notably cancer (Das and Singal, 2004; Sharma *et al*., 2010), and as such epigenetic therapies are being developed, which specifically target methylation (Yang *et al*., 2010).

Bisulfite treatment of DNA followed by next-generation sequencing provides quantitative methylation data with base pair resolution. Unmethylated cytosines are deaminated into uracils, which amplify as thymines during PCR (Krueger *et al*., 2012). Reads are then aligned to a reference genome, permitting changes of C to T. The resulting counts of cytosine and thymine at registered cytosine loci form the basis of further analysis. This general procedure has been adapted in various ways, with reduced representation bisulfite sequencing (RRBS) being one of the most widely used. RRBS involves using a restriction enzyme such as MspI (or TaqI) to cleave the DNA at CCGG (or TCGA) loci and selecting short reads for sequencing (Gu *et al*., 2011). This results in greater coverage of CpG dense regions at lower cost.

Several methods have been proposed to statistically test for differentially methylated region (DMRs). Almost all these methods perform a search for DMRs by testing individual cytosines followed by a post hoc aggregation procedure. Early methylation studies used Fisher's exact test (FET) to identify differentially methylated cytosines (DMCs) (Li *et al*., 2010; Challen *et al*., 2012). BSmooth (Hansen *et al*., 2012), one of the most widely used methods, performs local likelihood smoothing to generate methylation profiles for each sample, before testing individual locations in the profiles to identify DMCs. More recent methods, such as BiSeq (Hebestreit *et al*., 2013) and methylSig (Park *et al*., 2014), also employ local smoothing, together with a beta-binomial model of methylation at individual cytosines; both of these methods then aggregate the results of tests at individual loci to compute a measure of significance for DMRs. The beta-binomial method models biological variability at each cytosine location and hence requires a high replication level to achieve power. Coverage can also be problematic, as low coverage precludes statistical significance and high coverage can lead to over-confidence in calling DMRs, although the latter effect can be ameliorated by having a larger number of replicates. For instance, methylSig requires a minimum of three replicates per group and ignores loci which are covered by fewer than 10 reads by default. A recent method, MAGI (Baumann and Doerge, 2014), takes a different approach by testing directly for DMRs, rather than computing region-wide measures of significance from tests of individual cytosines. MAGI assumes the availability of genome-wide decision boundary methylation levels (which can be determined either from annotation or in a data-driven fashion). Methylation levels at each cytosine are then given a binary representation based on whether they exceed the decision boundary, and a single FET is performed over each region by counting how many cytosines have changed state.

**Fig. 1.**
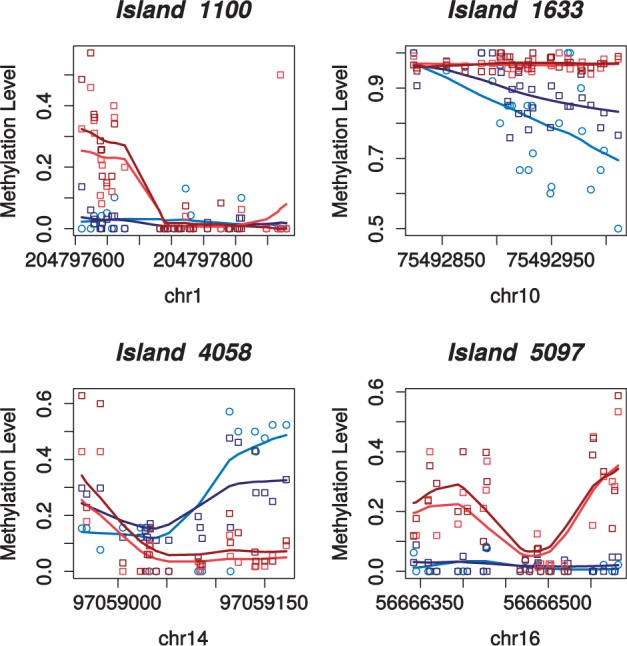
Methylation profiles of CpG clusters uniquely identified by the M^3^D method in a comparison of leukaemia (K562) and ESC cells, Sections 2.3 and 3.3. DMCs are not individually very different, yet the profile has changed shape in each case

Although these methods can be highly effective, no current method explicitly accounts for spatial covariation (MAGI implicitly assumes spatial homogeneity across a region). DNA methylation levels are often strongly spatially correlated: accounting for such correlations in a testing procedure could then lead to considerable increases in statistical power. Some examples of spatially correlated changes in the ENCODE data analysed in Section 3.2 are shown in Figure 1 and Supplementary Figure 1; notice that in all these examples, the change at individual cytosines is modest, and hence these regions would not be called as DMRs by currently existing methods. We remark that, although local smoothing methods like BSmooth (Hansen *et al*., 2012) attempt to capture spatial coherence, the local coherence is not an integral part of the testing procedure. Smoothing in this setting serves the dual purposes of filtering noise and highlighting large-scale changes in the methylation profile. Moreover, the shape of the methylation profile has been suggested as an important factor in predicting gene expression (VanderKraats *et al*., 2013), leading to a potentially functional role for methylation patterns. To our knowledge, there are no methods that test higher order properties, such as shape, of the methylation profiles over a region.

Here, we present maximum mean methylation discrepancy (M^3^D), a non-parametric statistical test for identifying DMRs from pre-defined regions, explicitly accounting for shape changes in methylation profiles. Our method is based on the maximum mean discrepancy (MMD), a recent technique from the machine learning literature, which tests whether two samples have been generated from the same probability distribution (Gretton *et al*., 2007, 2012). Similar non-parametric tests have already been applied to ChIP-Seq and RNA-Seq data (Schweikert *et al*., 2013; Drewe *et al*., 2013). Our contribution is to adapt the method for the specific challenges of bisulfite sequencing data, introducing an explicit control for confounding changes in coverage levels. Our method is used to test for changes in methylation profiles across regions, as opposed to individual cytosines, and we call as DMRs those regions whose variation cannot be explained by inter-replicate variability. We demonstrate the performance of M^3^D against existing methods on real and simulated data, showing a considerable increase in power and improved robustness against reduced replication and coverage levels.

## 2 Methods

The M^3^D method is designed to analyse aligned methylation data. Rather than testing individual cytosines and pooling them into putative DMRs, M^3^D considers changes in the methylation profile’s shape over a given region. To quantify shape changes, we compute the MMD over each region and adjust it to account for changes in the coverage profile across samples. Finally, we use a data-driven approach to compare test statistics based on the empirical likelihood of observing between-group differences among replicates. We restrict our analysis to CpGs only and combine data from both strands.

Selecting which regions to test is an important feature of a differential methylation study and must reflect the specific question being asked. Regions can either be pre-defined, such as a list of promoter regions, or generated from the data by selecting regions of dense CpGs (clusters) as in (Hebestreit *et al*., 2013). We keep this as a flexible option and instead focus on a general framework for region-based methylation analysis.

### 2.1 Maximum mean discrepancy

Formally, the MMD is defined as follows. Let F be a class of functions f:X→R over a metric space X with Borel probability measures *p*, *q*. We define the MMD as

(1) $$\text{MMD}[F,p,q]=\underset{f\in F}{\sup}(E_{p}[f(x)]-E_{q}[f(x)])$$

Intuitively, we are finding the mean over a bounded function that maximizes the difference between the probability distributions. For a sufficiently dense function class, this is equal to 0 if, and only if, *p* = *q*. Choosing F to be the unit ball in a reproducing kernel Hilbert space (RKHS) on X provides a searchable class of functions that retains this result (Gretton *et al*., 2007). For x,x′-independent random variables with distribution *p* and y,y′-independent random variables with distribution *q*, the square of the MMD becomes:

(2) $$\text{MM}{\text{D}}^{2}[F,p,q]=E_{p}[k(x,x\prime )]-2E_{p,q}[k(x,y)]+E_{q}[k(y,y\prime )]$$

In practice, for $X=\left\{x_{1},....,x_{m}\right\},Y=\left\{y_{1},\ldots ,y_{n}\right\}$ observations independently and identically distributed from *p* and *q,* respectively, we can compute a sample-based approximation to the MMD metric, giving rise to a feature representation in the RKHS, as

(3) $$\text{MMD}[X,Y,k]={\left[\frac{K_{xx}}{m^{2}}-\frac{2K_{xy}}{mn}+\frac{K_{yy}}{n^{2}}\right]}^{\frac{1}{2}}$$

$$\text{where}K_{xx}=\sum_{i,j=1}^{m}k(x_{i},x_{j}),K_{xy}=\sum_{i,j=1}^{m,n}k(x_{i},y_{j}),$$

$$\text{and}K_{yy}=\sum_{i,j=1}^{n}k(y_{i},y_{j})$$

### 2.2 The M^3^D statistic

We represent a RRBS dataset as a set of vectors *x_i_*, where each *x_i_* is composed of the genomic location of a cytosine *C_i_*, and the methylation status of that *C_i_* on one mapped read, $x_{i}=(C_{i},{\text{Meth}}_{i})$. Thus, there are as many *x_i_*s in a dataset as the number of mapped cytosines (within a CpG context). To define an MMD between datasets, we need to define a kernel function operating on pairs of vectors *x_i_*, *x_j_* to evaluate Equation (3). A natural choice is a composite kernel given by the product of a radial basis function (RBF) kernel on the genomic location and a string kernel on the methylation status:

(4) $$k_{\text{full}}(x_{i},x_{j})=k_{\text{RBF}}(x_{i},x_{j})k_{\text{STR}}(x_{i},x_{j})$$

(5) $$\text{Where}k_{\text{RBF}}(x_{i},x_{j})=\text{exp}[-{\left(C_{i}-C_{j}\right)}^{2}/2\sigma^{2}]$$

(6) $$\text{and}k_{\text{STR}}(x_{i},x_{j})=\{\begin{array}{cc} 1, & \text{if}{\text{Meth}}_{i}={\text{Meth}}_{j}\\ 0, & \text{otherwise} \end{array}$$

The RBF kernel, $k_{\text{RBF}}(x_{i},x_{j})$ retains spatial information at a scale determined by the hyper-parameter *σ*, which corresponds to the distance along the genome that displays methylation correlation. We model this parameter independently for each region, *R*, to reflect the local correlation structure, as $\sigma_{R}^{2}={\bar{x}}^{2}/2,\text{for}x\in R$, a heuristic suggested in (Gretton *et al*., 2012). Here, $\bar{x}$ refers to the median distance of all observations in region *R* across the datasets being compared. MMD distances computed using the above procedure would capture both differences in coverage profiles and differences in methylation profiles. A particular challenge of bisulfite sequencing data, and a central tenet of the RRBS procedure (Gu *et al*., 2011), is that the frequency with which a cytosine site is tested (the coverage) is unrelated to the methylation status. This poses a challenge in all bisulfite sequencing analysis, as the sampling distribution becomes a confounding factor in our attempt to understand methylation. We control for changes in the coverage profile by subtracting the analogous MMD of the coverage; the M^3^D metric is then given by:
(7)
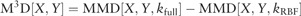

where *k*_full_ and 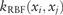 are as described in Equations (4) and (5) and the MMD terms are as in Equation (3). Henceforth, we refer to the first term, 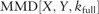 as the ‘full MMD’ and the second term, 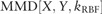, as the ‘coverage MMD’ for convenience.

The last term in Equation (7) represents the MMD of the data on a methylation-blind subspace. This implies that, in the large sample limit when the sample estimate of the MMD converges to the exact MMD of Equation (1), the M^3^D statistic is non-negative.

**Fig. 2.**
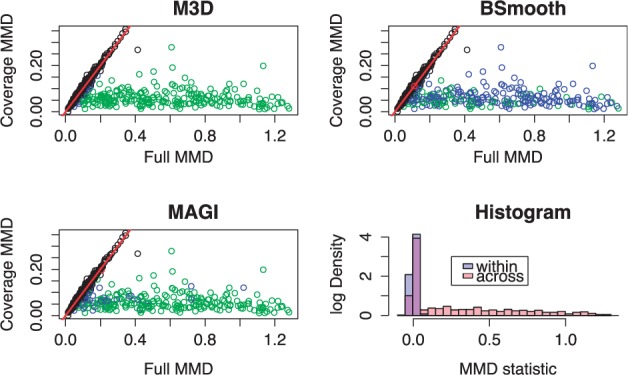
Simulation results. (**a–c**) We plot here the coverage MMD against the full MMD metric for all methods. The M^3^D test statistic is their difference, the distance in the x axis from the diagonal line. Each point is a CpG cluster, with black points being unchanged. DMRs are shaded according to whether they are called (M^3^D) or missed (BSmooth). (**a**) M^3^D identifies a clear relationship and calls almost all the clusters. (**b**) BSmooth calls some of the clusters but makes both types of error (Table 1). Classification bears little resemblance to the M^3^D method. (**c**) MAGI calls fewer regions, again with little semblance to the M^3^D method. (**d**) Histogram of test statistics for replicate values (blue) and with simulated changes (red), log scale

The M^3^D statistic will therefore be different from zero when there is a change in the methylation profile, independently of a change in the coverage profile. As a consequence, M^3^D between replicate RRBS experiments (which do not necessarily have identical coverage) should be close to zero or, equivalently, the full MMD should be equal to the coverage MMD. This is borne out in the data; the metrics strongly agree over replicates. Testing equality of metrics over 102 ENCODE RRBS datasets gives an *R*^2^ of 0.95. This can be seen in Supplementary Figure 2; specific examples can also be seen in Figures 2(a–c) and 4(a–c), where the dense region around the diagonal represents unchanged DMRs with M^3^D close to zero.

### 2.3 P-value calculation

We use the M^3^D as a test statistic by comparing the values observed across groups to those observed between replicates. We define our null distribution as the observed M^3^D values over all the testing regions between all replicate pairs. For a given region *r*, we compute the mean, *μ_r_* of the M^3^D values over all sample pairs across testing groups for *r*. The *P* value for *r* is the probability of observing *μ_r_* or higher among the null distribution. We use the Benjamini–Hochberg procedure to calculate false discovery rates (FDRs), rejecting clusters at a 1% significance level (Benjamini and Hochberg, 1995). Because each test corresponds to an entire region, this correction is less punitive than methods testing each cytosine location.

In general, we calculate the *P* value empirically. In order for the method to scale, we also provide a model-based approximation by fitting an exponential distribution to the 95th percentile of the null distribution. *P* values are calculated in the same manner using the fitted exponential. An example is shown in Supplementary Figure 3.

**Fig. 3.**
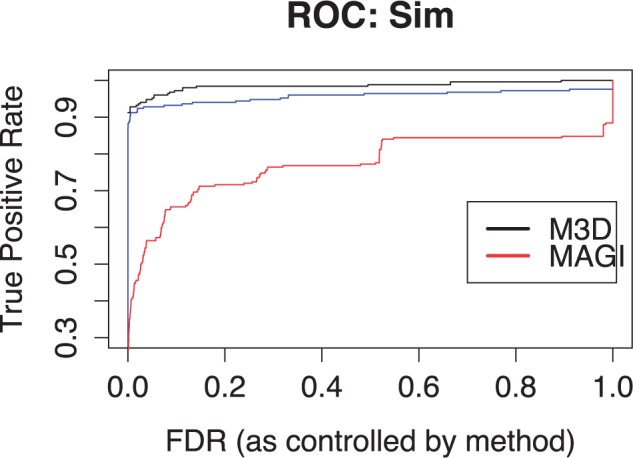
ROC curve. Here, we plot the true positive rate against the FDR for each method, reflecting the proportion of regions called at each FDR. From highest to lowest, we see M^3^D with empirical *P*-values, M^3^D with modelled *P*-values and MAGI. AUCs for the methods are 0.99, 0.96 and 0.77, respectively

At a given FDR cut-off, identifying DMRs amounts to identifying a threshold M^3^D value, *t*_fdr_ and calling all regions *r* with $\mu_{r}>t_{\text{fdr}}$. We find the empirical method to be marginally more accurate (Fig. 3) and report results with empirically calculated *P* values for the rest of this paper.

## 3 Datasets

We benchmarked the M^3^D method on a simulated dataset and two real datasets. We briefly describe here the two real datasets and the simulation procedure used. Summary statistics and figures of the testing regions are shown in Section 4 of the Supplementary Data.

### 3.1 Simulated data

To benchmark the ability of our method to detect true changes without introducing false positives, we resort to a simulation study. To simulate methylation profile changes with realistic statistics, we constructed our simulation from a real RRBS dataset.

We used an RRBS dataset of human embryonic stem cells, H1-hESC (described in the following section), consisting of two replicates. Dense CpG regions were identified using the procedure by Hebestreit *et al*., (2013), and for simplicity, we focused on the first 1000 on chromosome 1. We then simulated two more replicates to act as our testing group, as described in Section 5 of the Supplementary Data. The coverage statistics for the resulting dataset have a mean of 34.5 and a median coverage of 23 at each CpG site. We then selectively altered the methylation profile of randomly chosen regions in the simulated replicates to create known methylation changes and used the M^3^D method to test for DMRs.

To simulate methylation changes, we randomly selected 250 of the CpG clusters out of a possible 1000. We selected a short region within each cluster, at least 100-bp long and with a total coverage of at least 100, in each replicate. If necessary, we increased the size of the region until it occupied at least *n* CpG sites, where *n* was uniformly sampled from [4,20]. The methylation level, $L_{i}^{\text{old}}$, was calculated at each cytosine site, *C_i_* as the proportion of all the data points mapping to that site that were methylated. We measured the mean methylation of the sites and created a simulated methylation level, $L_{i}^{\text{new}}$, by hyper-methylating the region if it was <50% methylated on average and hypo-methylating it otherwise. The degree of methylation change was controlled by a parameter α∈[0,1], such that the new methylation level $L_{i}^{\text{new}}=(1-\alpha )L_{i}^{\text{old}}+\alpha$ if the region was being hyper-methylated and $L_{i}^{\text{new}}=(1-\alpha )L_{i}^{\text{old}}$ if it was being hypo-methylated. To vary the strength of methylation change, we tested the methods different values of alpha.

Simulated data were then created by sampling data points $\left\{x_{1},\ldots ,x_{n_{i}}\right\}$ at site *C_i_* with corresponding $\left\{{\text{Meth}}_{1},\ldots ,{\text{Meth}}_{n_{i}}\right\}$ sampled with probability $p({\text{Meth}}_{j}=\text{methylated})=L_{i}^{\text{new}}$, where *n_i_* is the coverage at location *C_i_*. Pseudocode for creating simulated data is shown in Section 5 of the Supplementary Data.

### 3.2 Human data

To test the M^3^D method on real data, we compared two Tier 1 tracks from the ENCODE consortium, GEO series GSE27584 (ENCODE Project Consortium *et al*., 2012). RRBS data from human embryonic stem cells, H1-hESC, were compared against leukaemia cells, K562. Both datasets were produced by the Myers Lab at the HudsonAlpha Institute for Biotechnology. The data are available pre-processed and aligned to the hg19 genome, and we used the resulting BED files. H1-hESC cells came from a human male and K562 from a female, so sex chromosomes were removed from the analysis. Testing regions were defined by clustering CpG sites in the same manner as for the simulations, and regions with no coverage in at least one sample were excluded from the analysis; this resulted in 14,104 genomic regions for testing.

To investigate the relationship between differential methylation and gene expression, we used the corresponding 200-bp paired end RNA-seq data for H1-ESC and K562 cells available in release 4 of the ENCODE consortium (ENCODE Project Consortium *et al*., 2012). Reads were aligned using TopHat and gene expression estimates in fragments per kilobase of transcript per million mapped reads (FPKM) were produced with Cufflinks (Trapnell *et al*., 2012). Gene expression estimates were averaged across the three replicates within each group and analysis was performed on the resulting changes across groups.

### 3.3 Mouse data

We compared a 4 replicate data RRBS data set from mouse strain B6C ESCs (GEO: GSE56572, Booth *et al*., 2014) to a 3 replicate data set consisting from sciatic nerve cells from postnatal day 10 (P10) mice (GEO: GSE45343, Varela-Rey *et al*., 2014). To define testing regions, we used the list of exons for Mus musculus provided by Ensembl in release 75 (Flicek *et al*., 2013). We excluded any exon regions with no coverage in one of the ESC cell samples or with <5 CpG sites in total, leaving 2359 regions in total. Again, data from both strands were combined. The median coverage at the remaining CpG loci was 24 for the ESCs and 11 for the P10 sciatic nerve cells.

## 4 Results

### 4.1 Simulations

We first benchmarked our method on a realistic simulated dataset generated as described in Section 3.1. Results were compared against BSmooth and MAGI. BiSeq and methylSig were also considered; however, because the dataset had lower replication than the minimum recommended by the authors, we decided not to use them. BSmooth was designed for whole-genome bisulfite sequencing (WGBS) data; to adapt the method to RRBS data, we followed the authors’ suggestion and altered the maximum allowable distance between neighbouring cytosines before smoothing (bioconductor mailing list: https://stat.ethz.ch/pipermail/bioconductor/2013-February/051020.html). Details for implementation of BSMooth and MAGI are provided in Section 6 of the Supplementary Data.

Figure 2 summarizes the results obtained with the methylation strength parameter *α* (see Section 3.1) set to 1. Of the 250 differently methylated regions, the M^3^D method called 232, with no falsely called DMRs. Figures 2(a–c) show scatterplots of coverage MMD on the *y* axis versus full MMD on the *x* axis for all 1000 regions, with colours denoting the results of the testing procedure using the different statistics. Individual regions are represented as circles, coloured according to whether the region was a true positive (green), a false positive (red), a false negative (blue) or a true negative (black). As discussed before, changes in methylation are likely to occur for regions that are mapped far from the diagonal. The figures show a clear cluster of regions about the diagonal (the unchanged regions) and a clearly identifiable group with much larger full MMD (the changed regions). Figure 2a shows the results of the testing procedure using the M^3^D statistic. As we see, M^3^D correctly identifies most of the 250 simulated changes. Receiver-operating characteristic (ROC) curves are shown in Figure 3. Note that BSmooth is omitted as the method does not test regions as a whole, rather identifies groups of DMCs within the region and hence does not output a relevant statistic for comparison.

We present the results of BSmooth and MAGI in same framework in Figure 2(b and c). BSmooth correctly called 67 of the regions with an additional 10 false positives, typically calling regions with similar coverage profile, which we expect is due to the effect of local likelihood smoothing. Figure 2b shows the BSmooth results; as we see, even regions with very marked shape differences (as quantified by M^3^D) were missed, which is to be expected as BSmooth does not include spatial correlations in the testing procedure. MAGI called 211 of the regions correctly, with two false positives (the FDR was set to 1%). Figure 2c shows that while many of the regions missed had a low M^3^D statistic, this was not always the case and there is not a simple relationship between the two methods indicating that the M^3^D method performs a genuinely different computation, as opposed to simply being more powerful. A histogram of the M^3^D test statistic is shown in Figure 2d for the replicates and the cross-group comparisons. The empirical testing distribution is shown in blue and is seen to be consistent around zero and sharply peaked. The simulated DMRs are easily distinguished.

We then investigated the sensitivity of our method by systematically altering the strength of the methylation changes using α=1,0.8,0.6 and 0.4. We compared the M^3^D method with BSmooth and MAGI and our results are listed in Table 1. The M^3^D method maintains a very creditable performance level for all the *α* values. This is due to the fact that neighbouring cytosines are being altered, and hence there are spatial correlations in the changes. Were the changes scattered randomly in the region M^3^D performance would weaken, whereas MAGI would remain robust. The sudden dip in performance of MAGI at α=0.4 is due to fewer of the cytosines’ methylation levels crossing the threshold value. It is remarkable that at all levels of *α* the use of the M^3^D statistic does not lead to any type I errors, though we note that the other methods have consistent type 2 error levels across these tests.

**Table 1.** Simulation results: sensitivity to low methylation changes

Alpha	1 (0.96, 0.10)			0.8 (0.76, 0.15)			0.6 (0.57, 0.16)			0.4 (0.38, 0.15)		
Meth change (mean, SD)												
	M^3^D	BS	MAGI	M^3^D	BS	MAGI	M^3^D	BS	MAGI	M^3^D	BS	MAGI
Correct	232	67	211	231	65	205	210	66	201	197	66	157
Type 1	0	10	2	0	7	2	0	10	2	0	10	0
Type 2	18	183	39	19	185	45	40	184	49	53	184	93

For various values of alpha, we show the corresponding mean and standard deviation of the methylation level (the total methylated reads divided by coverage at each CpG) change for the altered CpG sites and the results of testing the three methods. MMD outperforms BSmooth and MAGI.

To assess the sensitivity of the various methods to spatial correlations, we ran a further simulation, this time adding a ‘Gaussian bump’ of to the methylation profiles of the regions, at randomly chosen locations, with varying widths and strengths. Details are described in Section 5 of the Supplementary Data. Here, we found a more marked contrast in the performance of the methods, which we show in Table 2. ROC curves are shown in Section 7 of the Supplementary Data.

**Table 2.** Sim: Gaussian bump

	M^3^D	BS	MAGI
Correct	190	58	102
Type 1	0	11	1
Type 2	60	192	148

Results for adding a Gaussian bump to the methylation levels. Despite the overall change being smaller, M^3^D retains good performance by considering spatial correlations in the data.

### 4.2 Human data

We now describe the results of comparing two human datasets generated by the ENCODE consortium (see Section 3.2). We focus on three aspects: a general comparison of results between M^3^D and BSmooth, an analysis of the robustness of the results with respect to the coverage and an analysis of the functional relevance of our results.

### 4.3 DMR detection

**Fig. 4.**
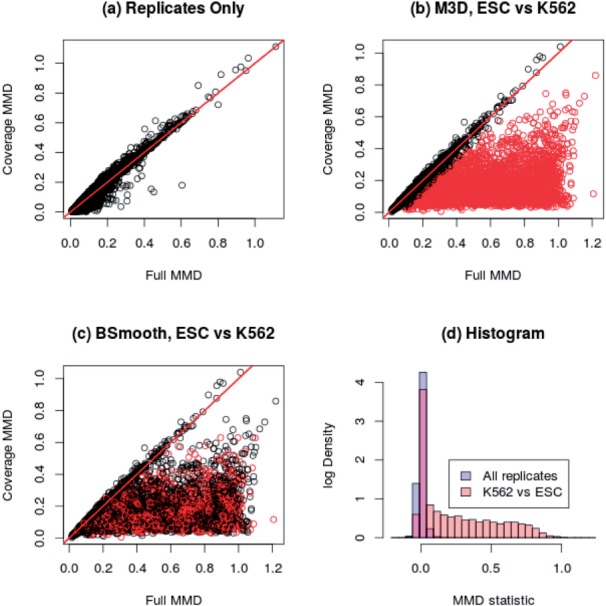
H1-hESC versus K562 Cells. Black dots are uncalled clusters, red are called. (**a**) Just the inter-replicate metrics are shown, for comparison with Figure 2. (**b**) Between-group clusters as called by M^3^D. (**c**) BSmooth identifies far fewer. Axes show the full and coverage MMD. Classification bears little resemblance to the M^3^D method. (**d**) The histogram of test statistics

Out of the 14,104 CpG regions selected for testing (see Section 3.2), M^3^D identified 4137 DMRs, and BSmooth and MAGI identified 1649 and 3101 DMRs, agreeing on 1328 and 2353 of the regions, respectively. In Figure 4, we present the results in the same form as Figure 2, where we again see that the M^3^D method produces genuinely different results. Figure 4a shows the method applied to the replicates only, where the methylation-blind and aware metrics agree. Figure 4(b and c) show the results of between-group testing by M^3^D and BSmooth, respectively. The data have a striking similarity to Figure 2a; this suggests that, on this real dataset, the M^3^D statistic provides an excellent measure of changes in methylation profiles. Similar to the simulated dataset, BSmooth does not behave in a consistent manner with respect to the M^3^D test statistic and many CpG clusters are missed (Fig. 4c). A histogram of the M^3^D test statistics is shown in Figure 4d, again confirming that the M^3^D statistics identifies a clear group of changed profiles between the two conditions. Comparisons to MAGI are shown in Section 8 of the Supplementary Data.

#### 4.3.1 Robustness to low coverage

To test the consistency of the M^3^D method to low coverage, we simulated a reduction in the coverage levels of the H1-hESC and K562 data. We discarded at random reads for the datasets to simulate a reduction in coverage by 75%, 50% and 25% in both datasets. The M^3^D method was used to find DMRs, and the results were compared across coverage levels; to alleviate the computational burden, we only considered CpG regions on chromosome 1.

**Fig. 5.**
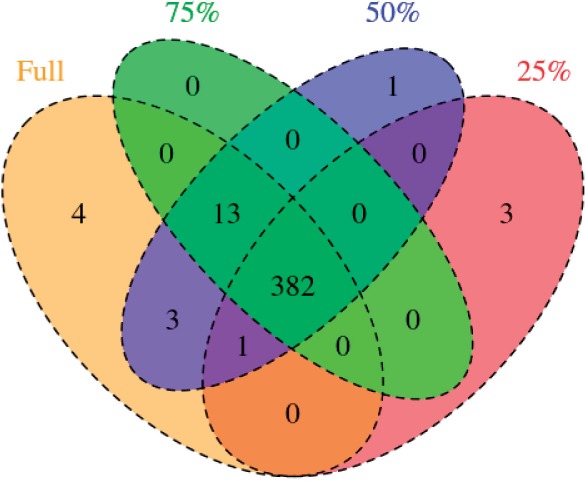
Venn diagram of calls with reduced coverage. Three-hundred eighty-two calls are consistent at all coverage levels. The method misses clusters at lower coverage levels, yet it does not call many DMRs that were not identified at higher coverage levels

Of the 1345 CpG clusters on chromosome 1, the M^3^D method identified 403 DMRs. Reducing coverage to 75%, 50% and 25% of the original level, the method identified 395, 399 and 386 DMRs, respectively, with 0, 1 and 1 DMRs not in the original set. To see the consistency of the calls, we show a Venn diagram in Figure 5. There is a strong overlap in the calls at all levels, indicating robustness in the method. Interestingly, although fewer regions are called at lower coverage levels, at each stage we do not see many new regions being called as might be expected. Although the empirical testing distribution is less accurate, the structure remains intact. Both BSmooth and MAGI showed similar consistency, although the number of regions called was lower in both cases. Results are shown in Section 9 of the Supplementary Data.

#### 4.3.2 Functional analysis of differential methylation regions

To investigate the functional relevance of our results, we interpreted the called DMRs in terms of the functional annotation and expression level of nearby genes. We used three sets of genomic regions, gene bodies, first exons and promoters; gene and exon regions were downloaded from Ensembl release 75 (Flicek *et al*., 2013) and promoter regions were defined as being 2000 bp upstream from the transcription start site. Tests were run on gene, promoter and first exon regions separately. We excluded regions with fewer than 5 CpGs and tested the remaining regions for changes in the methylation profiles using the M^3^D method.

As the M^3^D method provides a measure of the strength, but not direction of the methylation profile change, we measured the cross group expression change as the absolute value of the FPKM log-fold change and excluded all genes with <100 FPKM in one sample to avoid noise at low expression levels.

We identified 8747 gene body regions with sufficient expression and CpG content; among these, the M^3^D method called 404 as DMRs. We tested the absolute log-fold changes in expression between called and uncalled regions with a Wilcoxon rank-sum test and found the former was higher (*P* value: $2.2\times 10^{-16}$). Similarly, 103 of 4916 promoter regions were called as DMRs and showed an associated higher absolute log-fold expression change (Wilcoxon rank-sum test, *P* value: $1.18\times 10^{-6}$). Rather more first exon regions were tested, with 411 of 19 473 regions being called as DMRs. Again, there was an associated increase in the log-fold expression change (Wilcoxon rank-sum test, *P* value: $2.2\times 10^{-16}$). None of the 411 first exon DMRs were in the gene bodies of the gene region testing group, hence this is not a duplicate result. The median log-fold expression change for genes associated with uncalled regions was 0.15 for gene, promoter and first exon regions. In genes associated with called promoter regions, this rose to 0.24, and in genes and first exon regions, the median was 0.37 and 0.34, respectively (Supplementary Figure 12). These results support earlier studies outlining a stronger link between methylation in the first exon, as opposed the promoter region and gene expression (Brenet *et al*., 2011).

We performed an enrichment analysis for gene ontology (GO) terms using the Ontologizer software for the gene, promoter and first exon regions separately (Bauer *et al*., 2008). In each case, the population group was chosen to be the set of genes associated with the regions being tested, i.e. those with sufficient coverage and CpG counts, and the study group was the set of genes associated with the called regions. For this study, we examined the DMRs called by the M^3^D method against all the regions tested, independently of gene expression data. We used parent–child analysis and adjusted *P* values according with a Benjamini-Hochberg procedure at 10% FDR.

For the gene regions, we tested 2,692 called genes against 15,321 tested genes resulting in 208 GO terms being called. Among these, GO terms for embryonic morphogenesis, organ formation, growth, developmental growth, regulation of cell differentiation, pattern specification process and cell fate commitment were discovered, as might be expected in a comparison between ESCs and fully mature K562 cells. This suggests a connection between gene body methylation and cell function.

The first exon group was smaller, with 1,087 called gene associations in a population of 10,811. Thirty-one GO terms were statistically significantly enriched. Twenty-four of these terms were also enriched in the gene region analysis. Again, terms associated with cell differentiation, such as cell fate specification and cell fate commitment, were observed. This is striking, because these first exons were not in gene bodies of the gene testing regions.

The promoter group had 506 gene associations called out of a population of 8,114. Interestingly, we found no statistically significantly enriched GO terms in the analysis. The top 10 enriched GO terms, by statistical significance, are shown in Supplementary Tables 1, 2 and 3 in Section 11 for the three types of genomic regions tested.

### 4.4 Mouse data

To examine the robustness of the M^3^D statistic to changes in replication, we considered a comparison between two mouse datasets with larger replication, the ESCs dataset of (Booth *et al*., 2014) with four replicates and the neural dataset of Varela-Rey *et al*. (2014) with three replicates. Robustness to low replication is important; as remarked before, although many methods require at least three replicates in each dataset, many experimental protocols (including almost all the ENCODE RRBS data) provide only two replicates. We used the M^3^D method to identify DMRs with three and with two ESC replicates, and compared the set to those identified with the full four ESC replicate sets. DMRs were identified at a 1% FDR.

**Fig. 6.**
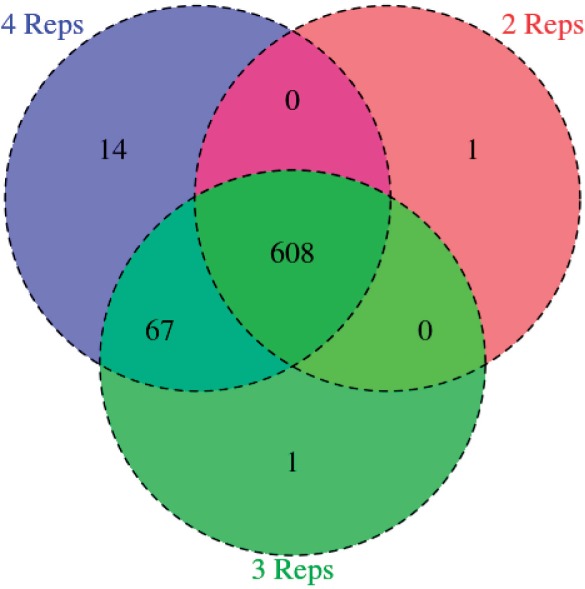
Venn diagram of calls with fewer replicates, for the case of four, three and two replicates for ESC cell control group

Of the 2359 exon regions tested, the M^3^Ds method identified 689, 676 and 609 with methylation profiles that differed significantly with respect to inter-replicate variation with 4, 3 and 2 replicates in the ESC group, respectively. As is shown in Figure 6, the overlap between the three sets of called regions accounts for almost 90% of the total. Importantly, although the testing lost power with lower replication (as can be expected), only one additional region was called, indicating that the method does not introduce many false positives with reduced replication levels.

### 4.5 Computing times

We report the running times for non-parallel implementations of all methods on the dataset used in Sections 3.2 and 4.3. This dataset represents a complete RRBS experiment consisting of 14,104 testing regions. On an ordinary desktop PC, MAGI took ∼30 s, BSmooth took almost 6 min (including smoothing) and M^3^D took ∼2 h. The M^3^D algorithm is linear in the number of testing regions and combinatorial in the number of replicates.

## 5 Discussion

We proposed the first kernel-based test for DMRs which exploits higher order spatial features of methylation profiles. Empirical comparisons on simulated and real data show a considerable increase in statistical power in comparison with the widely used BSmooth method (Hansen *et al*., 2012), as well as considerable robustness to low coverage and low replication. The M^3^D method also outperforms MAGI (Baumann and Doerge, 2014) in our simulations, as well as calling more DMRs in the real dataset, though this comes at a computational cost.

The increased power of the M^3^D approach is due to a number of factors. Firstly, the method is sensitive to spatially correlated changes in methylation profiles. Methylation profiles are known to be highly spatially correlated in general, and the results of our experiments imply that spatial correlation is also a feature of differences in methylation profiles between conditions, at least in the datasets considered. Secondly, the method explicitly accounts for differences in the coverage profiles between conditions, a confounding factor for other methods, as demonstrated in Figure 2. Thirdly, the method models inter-replicate variability on a regional basis along the whole genome. Each regional cross-group methylation change is compared with this distribution, and test statistics for each region represent how well the change in methylation profiles can be explained by inter-replicate variability. At present, other methods that consider inter-replicate variability do so on a CpG site-by-site basis, which lacks power with low replication and coverage and does not consider regional, spatial changes. When testing the method with different strengths of methylation change at CpG loci, we saw a sharp decrease in the number of regions being called as the methylation profile change over the regions became comparable to inter-replicate variability. Other methods experienced a less pronounced change in this regard.

Other studies have suggested that changes in shape of methylation profiles are important in predicting gene expression (VanderKraats *et al*., 2013). To test whether our method is able to capture functionally important changes in methylation profiles, we performed gene expression analysis with human data and showed a link between methylation changes called by the M^3^D method and gene expression changes between conditions. Further, the results support the hypothesis that gene expression is more closely linked to methylation in the first exon of a gene than to methylation in promoter regions (Brenet *et al*., 2011). GO analysis of first exon and whole gene methylation changes both revealed links to cell function, despite none of the exons overlapping the gene regions, a result that was not apparent for promoter methylation changes. Although our findings confirm a potential role for methylation profile shape as a predictor of gene expression, they do not provide biological mechanisms for linking methylation shape to gene expression and regulation. Although sequence variants and protein binding have been shown to be predictive of epigenetic variability (Gertz *et al*., 2011; Benveniste *et al*., 2014), we believe that further investigation of the mechanistic underpinnings of changes in methylation shape could be a valuable direction for research.

The M^3^D method provides a considerable increase in power over existing methods, yet it comes at the cost of computational intensity. In this study, we have restricted comparisons to datasets of low replication, as beta-binomial methods should prove effective with higher replication. We have also focused on sub-megabase scale changes for two reasons. Firstly, such an analysis is likely to be exploratory, in the sense that testing regions are not pre-defined, a key requirement for our method, and secondly, because BSmooth has proved adept at identifying large-scale changes in this setting and is computationally cheaper.

The M^3^D framework was developed with RRBS data in mind, yet, given its robustness to lower coverage, we expect that it may also be well suited for WGBS data. In the future, it will be interesting to develop models that explain the predictive power of methylation profiles in terms of other epigenetic marks.

## Supplementary Material

Supplementary Data
