# Supplementary material for: M3D: a kernel-based test for spatially correlated changes in methylation profiles
Source: Bioinformatics. 2014 Nov 13;31(6):809–16. doi: 10.1093/bioinformatics/btu749 (PMC4380032; doi:10.1093/bioinformatics/btu749)
Supplement: Supplementary Data [file supp_31_6_809__index.html]

M3D: a kernel-based test for spatially correlated changes in methylation profiles — M3D: a kernel-based test for spatially correlated changes in methylation profiles — Supplementary Data 

# M3D: a kernel-based test for spatially correlated changes in methylation profiles

## Supplementary Data

files

**Files in this Data Supplement:**

- Supplementary Data - pdf file
