# Supplementary material for: M3D: a kernel-based test for spatially correlated changes in methylation profiles
Source: Bioinformatics. 2014 Nov 13;31(6):809–16. doi: 10.1093/bioinformatics/btu749 (PMC4380032; doi:10.1093/bioinformatics/btu749)
Supplement: Supplementary Data [file supp_btu749_Supplementary_data.pdf]

Supplementary materials for the paper ”M^3D: a kernel-based test for spatially correlated changes in methylation profiles”

Tom Mayo, Gabriele Schweikert, and Guido Sanguinetti

October 21, 2014

1 Further Examples of Unique Calls

Further to the examples of regions uniquely called by the M^3D method in section 4.3 (Human Data) shown in Figure 1 in the main text, we include some more examples of shape based changes called by the method in Figure 1.

2 Equality of Metrics

Figure 2 shows a scatter-density plot of the MMD over each region for the coverage data against the full data for 102 ENCODE RRBS data sets. Testing equality of metrics gives an R^2 of 0.95, and we see some variation, representing inter-replicate variability.

3 Model approach to p-values

We fit an exponential distribution to the tail of the empirical distribution (the histogram of M^3D values between replicates), for the 95th percentile. This gives us a value for the exponential parameter λ, from which we calculate p-value, for a M^3D value of x by $e^{-\lambda x}$ (which is 1 minus the cumulative distribution function).

Figure 3 shows the result of the process. We take a conservative approach and hence over estimate the p-values.

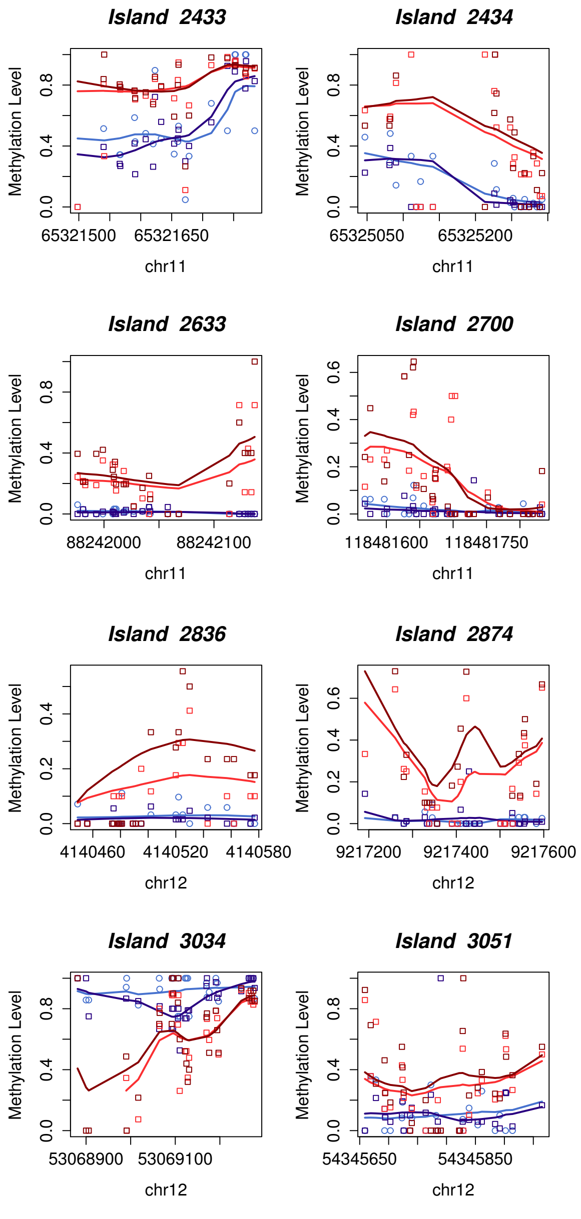

Figure 1: Examples of regions uniquely called by M^3D

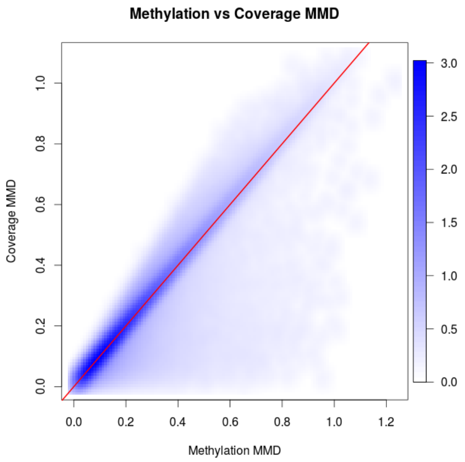

Figure 2: MMD over each region for the coverage kernel against the full kernel for 102 ENCODE RRBS data sets. There is strong agreement between the metrics.

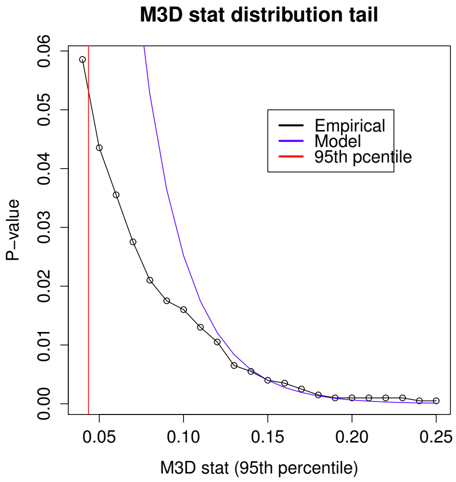

Figure 3: Modeling p-values. The black line shows the empirical distribution, the blue shows the exponential fit. The vertical red line indicates the 95th percentile.

4 Testing Regions

4.1 Simulation

Figure 4 shows histograms of the size of the testing regions for the Simulation.

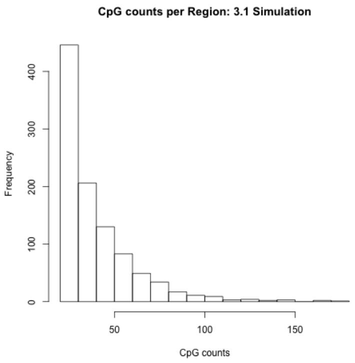

(a) CpG Counts

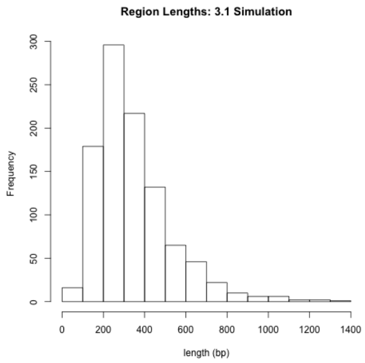

(b) Length of Regions

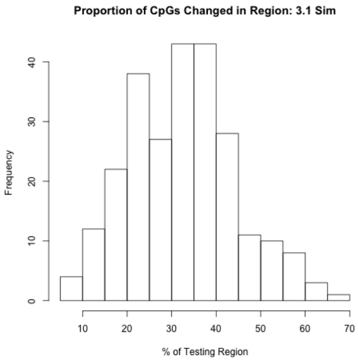

(c) % CpGs changed

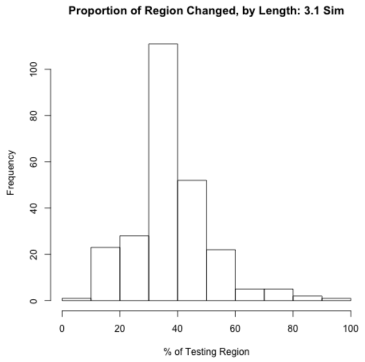

(d) % region changed by length

Figure 4: Statistics of the testing regions for the Simulation in sections 3.1 and 4.1

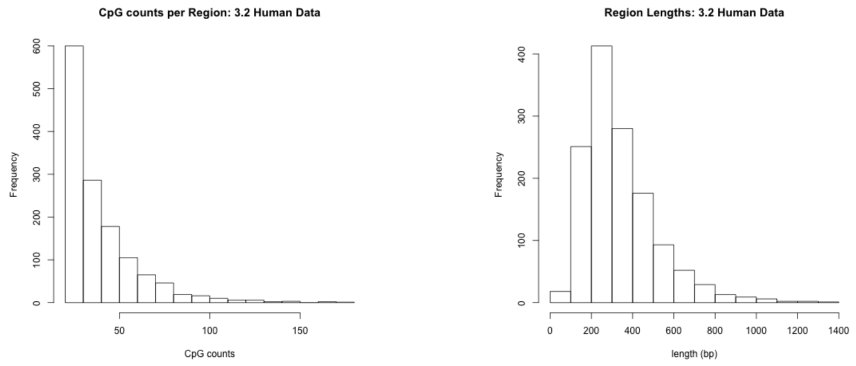

(a) CpG Counts (b) Length of Regions

Figure 5: Statistics of the testing regions for the Human data in sections 3.2, 4.2

4.2 Human Data

Figure 5 shows histograms of the size of the testing regions for the Human Data, used in sections 3.2 and 4.3.

4.3 Mouse Data

Figure 6 shows histograms of the size of the testing regions for the Mouse Data, used in section 3.3 and 4.4.

4.4 Testing Regions, Called vs Uncalled

In Figure 7, we show histograms of the region length and CpG count for regions that are called and not called by the M^3D method. The histograms in both cases have the same shape, indicating that there is unlikely to be a bias in the method for calling DMRs. The results come from section 4.3 (Human Data).

5 Simulation Details

The coverage profile was simulated on a region by region basis. For each region, r we sampled a 'total coverage multiple', t_r from $[0.8, 1.2]$ and a 'weighting variable' w_r from $[-0.3, 1.3]$. If we call the coverage profile of the

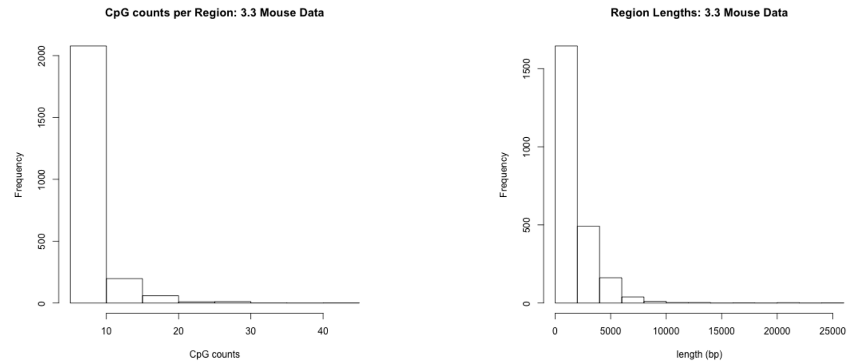

(a) CpG Counts

(b) Length of Regions

Figure 6: Statistics of the testing regions for the Mouse data in sections 3.3, 4.4

H1-hESC replicates $H1_1$ and $H1_2$ respectively, we created a coverage profile for the simulated replicate of region r by taking $w_r * H1_1 + (t_r - w_r) * H1_2$. This was performed for each simulated replicate.

A simulated replicate methylation profile was created by finding the mean methylation level of the H1 replicates at each site and multiplying that by the new coverage in each simulated replicate (and rounding to ensure integer values were maintained). In the event that the H1 replicates had low coverage (< 10 reads combined between them) we weighted the methylation level with a smoothed estimate of the neighbouring cytosines. The resulting coverage profiles had similar correlations to the inter-replicate values:

	Control1	Control2	Sim1	Sim2
Control1	1	0.88	0.84	0.84
Control2	0.88	1	0.87	0.87
Sim1	0.84	0.87	1	0.78
Sim2	0.84	0.87	0.78	1

Below we present the pseudocode describing how we simulated differential methylation in regions defined by the set *truths*, for a given value of α, which represents the strength of methylation change.

For the simulation involving adding Gaussian shaped bumps, we used

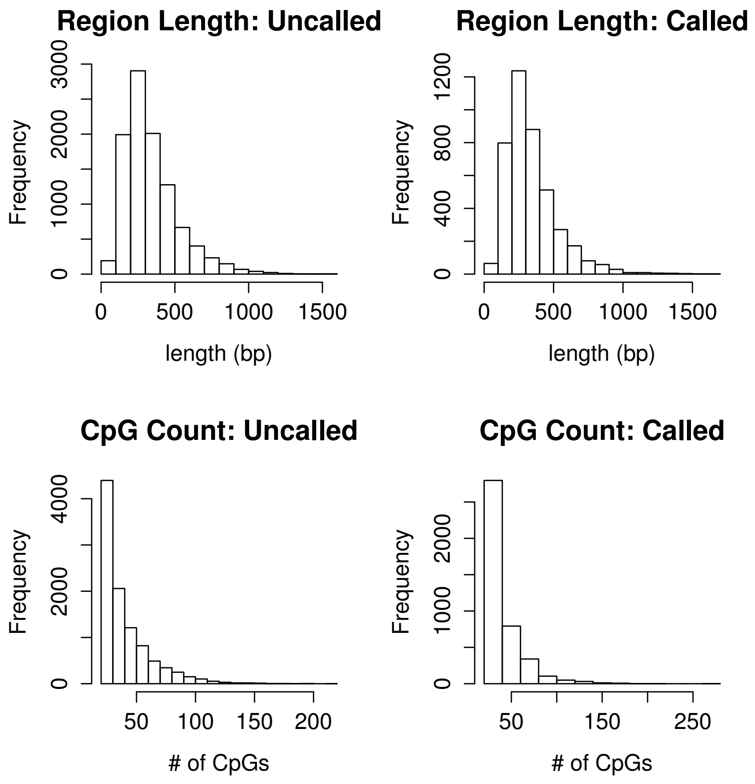

Figure 7: Statistics of the testing regions uncalled and called regions, from the Human Data (section 4.3). By region length (bp) and by CpG count.

Algorithm 1 Change the Methylation Profile of Regions in *truths*

for `region in truths` **do**
    `Randomly select start locus`
    while `CpGs in section` $<$ `4 OR section coverage` $<$ `100 OR section length` $<$ `100bp` **do**
        `Increase section by 1 locus on either side (within region)`
        if `length(section)` $>=$ `length(region)`$/3$ **then**
            `BREAK`
        end if
    end while
    for `CpG site` i `in section` **do**
        $L_i^{old} \leftarrow$ `methylation count/coverage at i`
        if $L_i^{old} <= 0.5$ **then**
            $L_i^{new} \leftarrow (1 - \alpha)L_i^{old} + \alpha$
        else
            $L_i^{new} \leftarrow (1 - \alpha)L_i^{old}$
        end if
        `methylation count at i` $\leftarrow$ `sample from` $\sim B($`coverage at i`$, L_i^{new})$
    end for
end for

the procedure in algorithm 2. σ, analogous to the variance in a normal distribution, controls the width of the change in bp, and was drawn uniformly from the integers in the interval [4,100], while the α parameter, representing the strength of the change was drawn from 0.6,0.8 and 1 with equal probability.

Algorithm 2 Gaussian Bump: Change the Methylation Profile of Regions in *truths*

for `region in truths` **do**
  `Randomly select start locus,` l
  for `CpG site` i `in section` **do**
    `Weight`$_i \leftarrow exp[-(i-l)^2/\sigma^2]$
    $L_i^{old} \leftarrow$ `methylation count/coverage at i`
    if `Weighted mean over region of` $L_i^{old} <= 0.5$ **then**
      $L_i^{new} \leftarrow L_i^{old} + \alpha \times Weight_i$
    else
      $L_i^{new} \leftarrow L_i^{old} - \alpha \times Weight_i$
    end if
    $L_i^{new} \leftarrow 0$ `or` 1 `if below or above resp.`
    `methylation count at i` $\leftarrow$ `sample from` $\sim B($`coverage at i`$, L_i^{new})$
  end for
end for

6 Comparison Methods: Details

BSmooth parameters were chosen to be as low as possible, whilst giving consistent performance across data sets, a minimum smoothing window (h) of 1000bp, a minimum methylation loci count (n) of 70, and a maximum gap between sites before smoothing is broken (maxGap) of 1000bp. These parameters were used for all data sets tested here.

MAGI was implemented as in the paper, using code kindly provided by the authors. The threshold value for each region was determined by k-means clustering with two clusters, as suggested in private correspondence from the authors.

An FDR of 1% was used for all methods, for all comparisons.

7 ROC curves

We present here the ROC curves for the simulation study using Gaussian bumps. We wish to determine whether pre-defined regions of interest are statistically significantly differentially methylated. MAGI takes the same approach, and hence a proper comparison is possible. BSmooth, on the other hand, is primarily a tool for discovering sections of the genome with differential methylation, relying on testing individual DMCs. The method then creates regions by chaining together CpGs with t-statistics greater than a threshold (determined from the data). This doesn't provide single, comparable statistics, and hence a direct comparison was not possible.

In Figure 9, we show the true positive rate against the FDR, as the method assumed it. Note, this isn't the false positive rate, but the attempt to control it. This gives us a sense of the power of the method to detect DMRs at various FDRs.

We found the are under the curves for the initial simulation to be 0.99, 0.96 and 0.77 for M^3D empirical, M^3D modelled and MAGI respectively. For the Gaussian simulation, these were 0.94, 0.84 and 0.77.

8 MAGI by M^3D statistics

In Figure 10a we plot the calls by MAGI with respect to the M^3D statistics, as with Figure 3 in the main text. As noted there MAGI misses approximately a quarter of the calls M^3D makes. These are shown in figure 10b. MAGI calls the regions with a higher M^3D statistic in general, but counterexamples indicate that a fundamentally different process is at work.

9 BSmooth & MAGI with low Coverage

Figures 11a and 11b show the results of the test for reduced coverage for BSmooth and MAGI respectively.

10 Expression Fold Changes

Figure 12 shows histograms for the absolute log-fold change of the called and uncalled regions for genes, first exons and promoters respectively. All three show an increase, but it is more pronounced for gene regions and first exons.

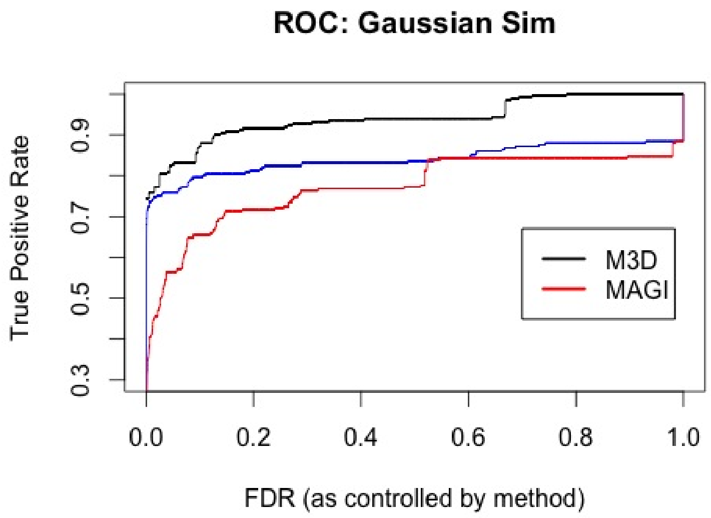

Figure 8: Gaussian Sim

Figure 9: ROC curve for Gaussian simulation. Here we plot ROC curves of the true positive rate against the controlled FDR for M^3D and MAGI. Black is the empirical p-values from M^3D, blue is the modelled p-values, and red is MAGI.

11 Gene Ontology Terms

Tables 1, 2 and 3 contain the top ten enriched gene ontology terms for the H1-hESC cells vs K562 cell comparison study, for gene regions, first exons and promoter regions in Tables 1, 2 and 3 respectively. We include the Benjamini-Hochberg adjusted p-values. Note that none of the promoter region terms achieve statistical significance, while the gene and first exon terms show a high degree of overlap.

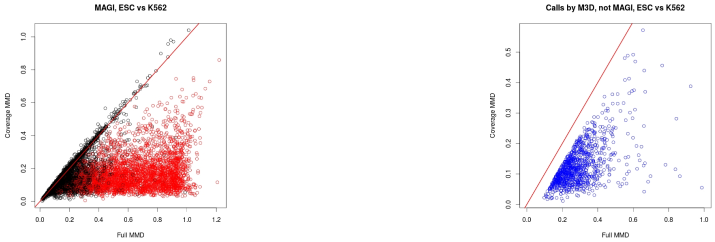

(a) MAGI (b) Calls by M^3D, not MAGI

Figure 10: MAGI, Human Data. As with Figure 3 in the main text, we plot the coverage MMD against the full MMD metric . The M^3D test statistic is their difference, the distance in the x-axis from the red line. Each point is a CpG cluster. Black are unchanged, Green are correctly called DMRs, Blue are missed DMRs, Red are incorrectly called clusters. In (b) we show the regions called by M^3D but not MAGI.

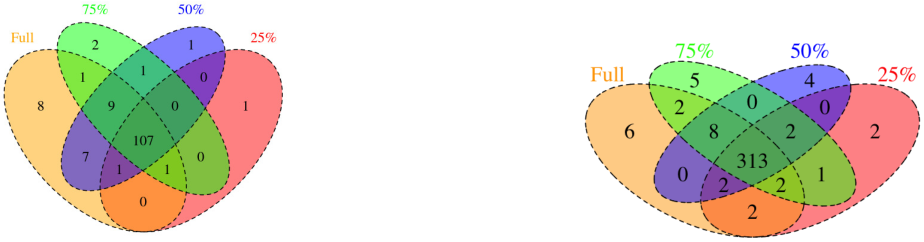

(a) BSmooth (b) MAGI

Figure 11: Venn Diagram of Calls with Reduced Coverage. (a-b) The methods show broadly similar consistency to the M^3D method with respect to lower coverage.

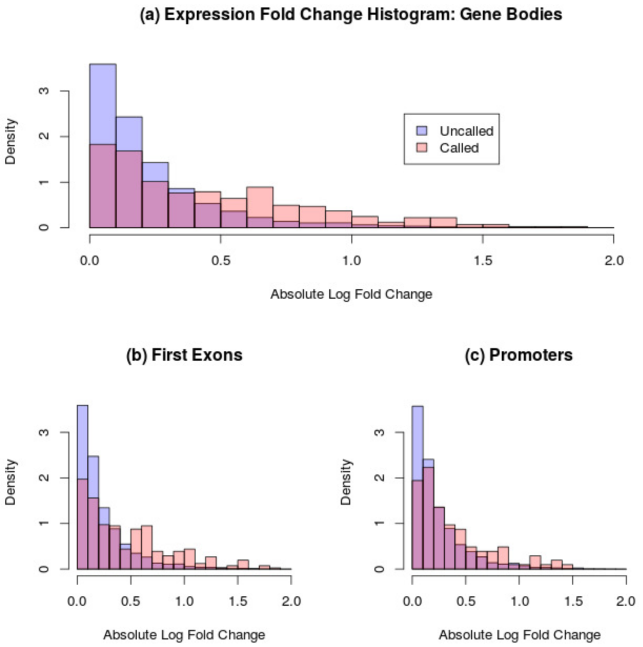

Figure 12: Absolute expression log-fold changes for called regions (red) and uncalled regions (blue) for (a) gene regions, (b) first exon regions and (c) promoter regions.

Table 1: GO terms: Gene Regions

	ID	name	p.adjusted
1	GO:0071944	cell periphery	1.437708223467503E-25
2	GO:0005886	plasma membrane	6.444067866565821E-22
3	GO:0044459	plasma membrane part	4.062219312427493E-20
4	GO:0032501	multicellular organismal process	3.1909227117764803E-13
5	GO:0044425	membrane part	4.4083353990865556E-13
6	GO:0003677	DNA binding	5.045748555333859E-13
7	GO:0043565	sequence-specific DNA binding	9.122745220828804E-11
8	GO:0044707	single-multicellular organism process	9.790514431161451E-11
9	GO:0004872	receptor activity	1.6558910963111396E-10
10	GO:0022610	biological adhesion	1.6465615260421583E-9

Table 2: GO terms: First Exons

	ID	name	p.adjusted
1	GO:0071944	cell periphery	1.2919365432790091E-5
2	GO:0005886	plasma membrane	5.622471574689E-5
3	GO:0003677	DNA binding	0.0012341786928683266
4	GO:0032501	multicellular organismal process	0.004089560778137887
5	GO:0044459	plasma membrane part	0.0067889743969307675
6	GO:0045165	cell fate commitment	0.0067889743969307675
7	GO:0044707	single-multicellular organism process	0.0067889743969307675
8	GO:0007010	cytoskeleton organization	0.0067889743969307675
9	GO:0022610	biological adhesion	0.00819729130007154
10	GO:0051965	positive regulation of synapse assembly	0.015205197324440567

Table 3: GO terms: Promoter Regions

	ID	name	p.adjusted
1	GO:1990351	transporter complex	0.4262394723222016
2	GO:0006351	transcription, DNA-templated	0.5120415178222222
3	GO:0010977	negative regulation of neuron projection development	0.5261134605589649
4	GO:0044459	plasma membrane part	0.6876369065744885
5	GO:0003677	DNA binding	0.6876369065744885
6	GO:0033764	steroid dehydrogenase activity	0.6876369065744885
7	GO:0007260	tyrosine phosphorylation of STAT protein	0.6876369065744885
8	GO:0006811	ion transport	0.6876369065744885
9	GO:0019825	oxygen binding	0.6876369065744885
10	GO:0080090	regulation of primary metabolic process	0.6876369065744885
